# Indulging Curiosity: Preliminary Evidence of an Anxiolytic-like Effect of Castor Oil and Ricinoleic Acid

**DOI:** 10.3390/nu16101527

**Published:** 2024-05-18

**Authors:** Khalin E. Nisbett, Leandro F. Vendruscolo, George F. Koob

**Affiliations:** 1 Graduate Program in Neuroscience, Graduate College, University of Illinois Chicago, Chicago, IL 60607, USA; 2 Stress and Addiction Neuroscience Unit, Integrative Neuroscience Research Branch, National Institute on Drug Abuse Intramural Research Program and National Institute on Alcohol Abuse and Alcoholism Division of Intramural Clinical and Biological Research, National Institutes of Health, Baltimore, MD 21224, USA; 3 Neurobiology of Addiction Section, Integrative Neuroscience Research Branch, National Institute on Drug Abuse Intramural Research Program, National Institutes of Health, Baltimore, MD 21224, USA

**Keywords:** anxiety, kolliphor, castor oil, ricinoleic acid

## Abstract

In the process of validating the elevated zero maze, a common test of anxiety-like behavior, in our laboratory, we demonstrated an anxiolytic-like effect of castor oil and its primary component, ricinoleic acid. We tested the effects of vehicle and chlordiazepoxide in male mice in the elevated zero maze following a 30-min pretreatment time. Chlordiazepoxide is a United States Food and Drug Administration-approved drug that was previously shown to exert anxiolytic-like effects in both the elevated zero maze and elevated plus maze. Chlordiazepoxide was administered at doses of 5 or 10 mg/kg. We used 5% polyoxyl 35 castor oil (Kolliphor^®^ EL) and saline as treatment vehicles and found that the effect of chlordiazepoxide on open zone occupancy and open zone entries was blunted when 5% Kolliphor was used as the vehicle. These tests demonstrated that chlordiazepoxide increased open zone occupancy and entries in the elevated zero maze more effectively when saline was used as the treatment vehicle and that Kolliphor dampened the anxiolytic-like effect of chlordiazepoxide when it was used as the treatment vehicle. Notably, 5% Kolliphor alone slightly increased baseline open zone occupancy and entries. Given that Kolliphor is a derivative of castor oil, we next tested the effect of 5% castor oil and 5% ricinoleic acid, which is a major component of castor oil. We found that both castor oil and ricinoleic acid increased open zone occupancy but not entries compared with saline. Altogether, our findings demonstrate that Kolliphor, castor oil, and ricinoleic acid may exert anxiolytic-like effects in male mice in the elevated zero maze. This potential anxiolytic-like effect of castor oil is consistent with its well-established beneficial effects, including anti-inflammatory, antioxidant, antifungal, and pain-relieving properties.

## 1. Introduction

Anxiety and mood disorders are increasingly prevalent [1]. Within the past decade, rates of diagnoses of these disorders have increased significantly. Approximately 18% of the US adult population currently meets the *Diagnostic and Statistical Manual of Mental Disorders*, 5th edition, criteria for one of these disorders annually [1]. The development of pharmacotherapeutics for these disorders is slow and cumbersome. Accessibility to approved medications is often limited, and their efficacy and side effect profiles are not ideal. For many decades, phytotherapy was incorporated into various cultures worldwide. The authors recall children being regularly nourished with herbal teas in Nevis (KEN) and Brazil (LFV) and castor oil (*Ricinus communis* L.) in the northeastern United States (GFK), among other plant-based remedies for various mild acute illnesses. These practices are less common now, and research on their therapeutic efficacy and mechanisms of action is limited. We recently performed a series of experiments to validate the elevated zero maze in our laboratory. We observed the potential anxiolytic-like effects of castor oil and its main component, ricinoleic acid, in mice. These preliminary findings suggest that castor oil may have neuroprotective properties, but this warrants further investigation.

Although the origins of the castor bean plant have been widely debated, its numerous beneficial properties have been well-known throughout the world [2]. Previous studies have also shown that castor oil and other derivatives of the castor bean plant are safe to use in topical and oral applications [3]. Castor oil is predominantly composed of fatty acids and a small percentage of tocopherols (i.e., α-, β-, γ-, and δ-tocopherol) [4]. Its predominant fatty acid component is ricinoleic acid (12-hydroxyoleic acid), which comprises 75–95% of castor oil. It also contains a large proportion of triricinolein, the fatty acid ester (triglyceride) of ricinoleic acid [4].

Castor oil was used as early as 500 BC by the ancient Egyptians in ointments (unguent), in 18th century Europe for skin healing, and across the world for its various medicinal benefits, such as treating inflammatory, dermatological, cardiovascular, oncological, and ophthalmologic illnesses. Scientific studies support its antifungal [5,6,7], antimicrobial [8], antiviral [9], wound healing [10,11,12], and analgesic [13,14] properties. It has also been used as a delivery vehicle and an additive for topical, transdermal, and oral drugs [15].

As an oral preparation, castor oil has been and continues to be used most commonly as a laxative (15–60 mL) [16,17] and a labor-inducing agent [18] in the Western world. Also, a study demonstrated the significant efficacy of castor oil as an analgesic in patients with osteoarthritis at doses of 2.7 mL/day for 4 weeks in a randomized, double-blind comparative clinical trial [13]. Castor oil mixed with parsley, *Cajanus indica*, or other oils is orally administered for the treatment of asthma, cerebral congestion, and psychiatric conditions [2,19,20]. Other decoctions of the castor bean plant are ingested to treat respiratory, digestive, osteopathic, urogenital, infectious, and obstetric conditions [2]. However, care should be taken in preparing castor oil because the castor bean from which it is extracted also contains a phytotoxin, ricin, that may be harmful when ingested.

Castor oil and its derivatives are also used in the food industry, most commonly as food additives (e.g., as emulsifiers) [21,22]. Ricinoleic acid, the major component of castor oil, is an unsaturated omega-9 fatty acid and a hydroxy acid. It is also found in numerous foods and vegetable and seed oils including olive oil, grapeseed oil, and cottonseed oil [23], which are major components of Mediterranean and Western diets. Like castor oil, ricinoleic acid is approved for daily human intake at doses of 0–0.7 mg/kg by the Joint Food and Agriculture Organization/World Health Organization Expert Committee on Food Additives [3].

Herein, we describe our findings chronologically to highlight instructive aspects of this study. To validate the elevated zero maze in mice in our laboratory, we tested the effects of vehicle and a benzodiazepine, chlordiazepoxide, in male mice following a 30-min pretreatment time. Chlordiazepoxide is a United States Food and Drug Administration (FDA)-approved anxiolytic drug that has anxiolytic-like effects in the elevated zero maze and elevated plus maze in rodents [24,25]. We administered chlordiazepoxide at doses of 5 and 10 mg/kg and used 5% polyoxyl 35 castor oil as the treatment vehicle. Polyoxyl 35 castor oil is trademarked as Kolliphor^®^ EL (formerly Cremaphor^®^ EL). It is referred in this manuscript as Kolliphor. Kolliphor is a synthetic, nonionic surfactant that can function as a solubilizer and is a synthetic derivative of castor oil [2]. It is commonly used as an emulsifying and solubilizing agent to prepare chemicals for use in preclinical research and pharmaceutical and cosmetic products.

## 2. Materials and Methods

### 2.1. Study Approval

This study was conducted in accordance with the National Institutes of Health Guide for the Care and Use of Laboratory Animals and approved by the National Institute on Drug Abuse Intramural Research Program Animal Care and Use Committee (20-INRB-27; 8 September 2020).

### 2.2. Animals

One hundred and two male C57Bl/6J mice, weighing 25–30 g at the start of testing, were used. They were purchased from Jackson Laboratory (Bar Harbor, ME, USA) and acclimated to the laboratory for at least 1 week before the experiments began. The mice were housed in same-sex groups (2–4 per cage) in plastic cages (28 cm width × 17 cm length × 12 cm height) with free access to food and water except during the experimental procedures. The mice were kept in a room with a 12 h/12 h light/dark cycle (lights on at 7 AM) with controlled temperature (22 °C ± 2 °C) and humidity (50–60%). Behavioral testing occurred during the light cycle following previously reported experimental conditions that allow for the detection of anxiolytic- and anxiogenic-like effects [26,27]. The mice were randomly assigned to experimental groups. Sample sizes (*n* = 8–16) were determined based on our previous publications to ensure adequate power to detect statistical differences [26,27]. No animals were excluded from the study.

### 2.3. Drug Administration

#### 2.3.1. Reagents and Materials

Saline was obtained from Baxter Healthcare Corporation (Deerfield, IL, USA), Kolliphor from Sigma-Aldrich (St. Louis, MO, USA), castor oil and ricinoleic acid from Frontiers Co-op (Norway, IA, USA), and chlordiazepoxide from the NIDA Drug Supply Program (Bethesda, MD, USA).

#### 2.3.2. Experiment 1

Vehicle (10 mL/kg 5% Kolliphor in saline) or chlordiazepoxide (5 and 10 mg/kg) was administered subcutaneously 30 min before testing in the elevated zero maze in male mice. Kolliphor at a concentration of 5% in solution (saline) is well-tolerated across numerous species and is a common concentration that is used to dissolve pharmaceuticals [28,29]. Three experimental groups were tested: vehicle (*n* = 8), 5 mg/kg chlordiazepoxide (*n* = 8), and 10 mg/kg chlordiazepoxide (*n* = 8).

#### 2.3.3. Experiment 2

Vehicle (10 mL/kg saline) or chlordiazepoxide (5 and 10 mg/kg) was administered subcutaneously 30 min before testing in the elevated zero maze in male mice. Three experimental groups were tested: vehicle (*n* = 8), 5 mg/kg chlordiazepoxide (*n* = 8), and 10 mg/kg chlordiazepoxide (*n* = 8).

#### 2.3.4. Experiment 3

Saline, 5% Kolliphor, 5% castor oil, or 5% ricinoleic acid was administered subcutaneously 30 min before testing in the elevated zero maze in male mice. The solvent for Kolliphor, castor oil, and ricinoleic acid was saline. Four experimental groups were tested: vehicle (*n* = 12), 5% Kolliphor (*n* = 14), 5% castor oil (*n* = 16), and 5% ricinoleic acid (*n* = 14).

### 2.4. Elevated Zero Maze

The elevated zero maze test was conducted as previously reported [27]. The zero maze has four quadrants (two open quadrants with 1 cm high translucent edges that were separated by two closed quadrants with 15 cm high opaque walls; lanes were 5 cm wide). Mice were allowed to acclimate to the experimental room for at least 1 h prior to testing. Testing began after the administration of treatment and when the experimenter placed the mouse in either of the open zones. Each mouse was allowed to explore the elevated zero maze for 5 min, and behavior was recorded using a wall-mounted Stoelting USB camera (Wood Dale, IL, USA). Each mouse was tested once. The experimenter was aware of group allocation prior to the start of the testing; however, all measurements were recorded automatically using the video software. Behavior was analyzed in real time by AnyMaze tracking software (Wood Dale, IL, USA). The percent time that the animal spent in the open quadrants (open zone occupancy) and number of entries into the open quadrants (open zone entries) were used as measures of anxiety-like behavior. Increases in open zone occupancy and entries were interpreted as a reduction of anxiety-like behavior. Testing occurred in a dimly lit room (55 ± 15 lux) between 8 AM and 5 PM. We used the elevated zero maze because of its established predictive validity (i.e., FDA-approved anxiolytic drugs increase open zone occupancy and entries in mice in this task) [30].

### 2.5. Statistical Analysis

The statistical analyses were conducted using Prism software (GraphPad, San Diego, CA, USA). All data met the assumption of a normal distribution for statistical tests, and variance was similar between groups. The data were analyzed using one-way analyses of variance (ANOVAs) to determine the effect of treatment. ANOVAs that yielded a significant effect of treatment were followed by Holm–Sidak’s multiple-comparison post hoc tests. Values of *p* < 0.05 were considered statistically significant for all tests. Effect sizes (Cohen’s d) for each experimental group were calculated using Microsoft Excel Version 16.85 and reported.

## 3. Results

**Figure 1:**
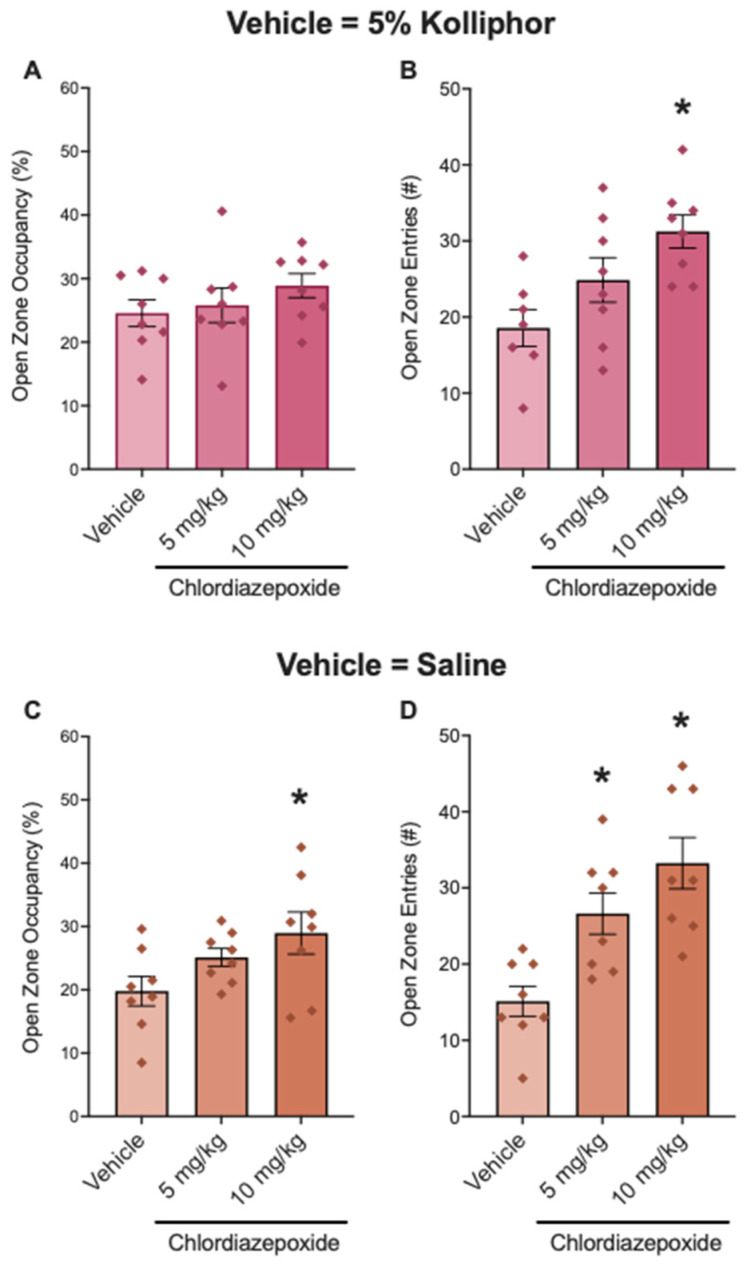
Chlordiazepoxide reduced open zone occupancy in male mice in the elevated zero maze when saline, but not 5% Kolliphor, was used as the vehicle. Chlordiazepoxide did not increase open zone occupancy (**A**) but increased open zone entries (**B**) in the elevated zero maze when 5% Kolliphor was used as the vehicle. The plots show data for male mice that were treated with saline (control group; *n* = 8), 5 mg/kg chlordiazepoxide (*n* = 8), and 10 mg/kg chlordiazepoxide (*n* = 8) in 5% Kolliphor. Chlordiazepoxide increased open zone occupancy (**C**) and entries (**D**) in the elevated zero maze when saline was used as the vehicle. The plots show data for male mice that were treated with saline (control group; *n* = 8), 5 mg/kg chlordiazepoxide (*n* = 8), and 10 mg/kg chlordiazepoxide (*n* = 8) in saline. * *p* < 0.05, difference from saline.

We examined the effect of vehicle (5% Kolliphor in saline) or chlordiazepoxide in the elevated zero maze, see Figure 1A,B. The ANOVA demonstrated a main effect of treatment on open zone entries (*F*_2,20_ = 6.085, *p* = 0.0086) (Figure 1B) but no main effect of treatment on open zone occupancy (*F*_2,21_ = 0.9623, *p* = 0.9623) (Figure 1A). Post hoc tests demonstrated that 10 mg/kg (*p* = 0.0047, d = 2.023) but not 5 mg/kg (*p* = 0.0985, d = 0.8594) chlordiazepoxide increased open zone entries. Effect sizes (d) for open zone occupancy (when 5% Kolliphor was used as the vehicle) are as follows: d = 0.1809 for 5 mg/kg chlordiazepoxideand d = 0.7631 for 10 mg/kg chlordiazepoxide.

We then examined the effect of vehicle (saline) and chlordiazepoxide in the elevated zero maze, see Figure 1C,D. The ANOVA demonstrated the main effects of treatment on open zone occupancy (*F*_2,21_ = 3.459, *p* = 0.0503) (Figure 1C) and entries (*F*_2,21_ = 11.19, *p* = 0.0005) (Figure 1D). Post hoc tests demonstrated that 10 mg/kg (*p* = 0.0318, d = 1.150) but not 5 mg/kg (*p* = 0.1426, d = 1.009) chlordiazepoxide increased open zone occupancy and that both 5 mg/kg (*p* = 0.0074, d = 1.7376) and 10 mg/kg (*p* = 0.0003, d = 2.404) chlordiazepoxide increased open zone entries.

**Figure 2:**
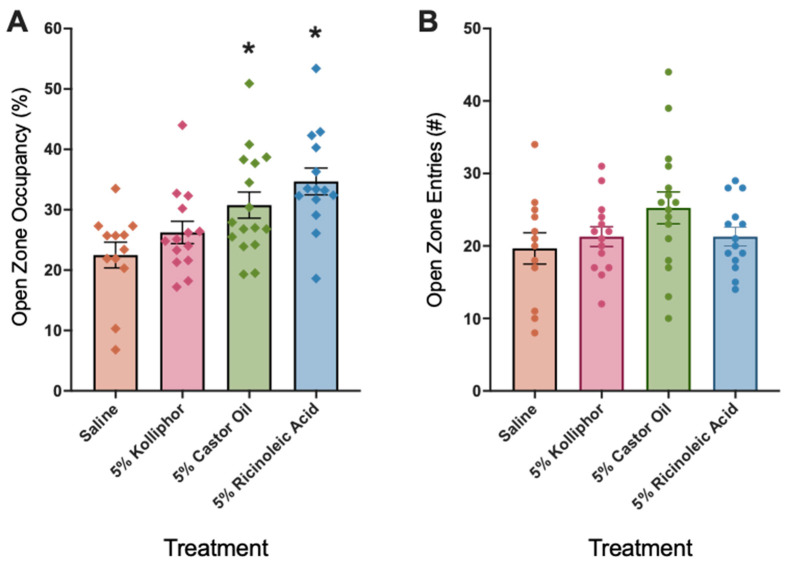
Five percent (5%) castor oil and 5% ricinoleic acid increased open zone occupancy (**A**) but not open zone entries (**B**) in the elevated zero maze in male mice. The plots show data for male mice that were treated with saline (control group; *n* = 12), 5% Kolliphor (*n* = 14), 5% castor oil (*n* = 16), and 5% ricinoleic acid (*n* = 14). * *p* < 0.05, difference from saline.

Given that male mice that were treated with 5% Kolliphor exhibited slightly higher open zone occupancy and entries compared with male mice that were treated with saline and that Kolliphor is a derivative of castor oil, we tested anxiolytic-like effects of 5% Kolliphor, 5% castor oil, and 5% ricinoleic acid, see Figure 2A,B. Ricinoleic acid is a major component of castor oil. The ANOVA demonstrated a main effect of treatment on open zone occupancy (*F*_3,52_ = 5.973, *p* = 0.0014) (Figure 2A) but not open zone entries (*F*_3,52_ = 1.788, *p* = 0.1609) (Figure 2B). Post hoc tests demonstrated that 5% castor oil (*p* = 0.0165, d = 1.032) and 5% ricinoleic acid (*p* =0.0008, d = 1.552) but not 5% Kolliphor (*p* = 0.2331, d = 0.5241) increased open zone occupancy compared with saline. Effect sizes (d) for the open zone entry measures are as follows: d = 0.2577 for 5% Kolliphor, d = 0.6865 for 5% castor oil, and d = 0.2642 for 5% ricinoleic acid.

## 4. Discussion

The present results showed that castor oil and ricinoleic acid reduced anxiety-like behavior in male mice. Notably, 5% Kolliphor increased baseline open zone occupancy and entries, and 5% castor oil and 5% ricinoleic acid increased open zone occupancy but not entries compared with saline.

We also found that chlordiazepoxide increased open zone entries but not open zone occupancy when 5% Kolliphor was used as the vehicle, which was partially inconsistent with previous studies that used saline as the vehicle [24,25], an effect that was potentially attributable to an increase in exploration of the open zones by Kolliphor itself. As such, we repeated the experiment using saline as the vehicle and found that chlordiazepoxide increased both open zone occupancy and entries. These validation studies demonstrated that chlordiazepoxide reduced anxiety-like behavior when saline or 5% Kolliphor was used as the vehicle, but the effect was less pronounced with Kolliphor because of slightly higher baseline open zone occupancy and entries in these groups. Another potential explanation for the Kolliphor-induced blunting of chlordiazepoxide’s anxiolytic-like effect is that it may affect the disposition of various drugs by changing unbound drug concentrations through micellar encapsulation [31]. However, these results also suggest that vehicles that are commonly used in preclinical studies need to be more rigorously tested in behavioral studies.

Given that male mice that were treated with 5% Kolliphor exhibited slightly higher open zone occupancy and entries compared with male mice that were treated with saline and that Kolliphor is a derivative of castor oil, we tested anxiolytic-like effects of 5% Kolliphor, 5% castor oil, and 5% ricinoleic acid. Ricinoleic acid is a major component of castor oil. The average dose of castor oil used in this study was 0.5 mL/kg, which is 40 times lower than the laxative dose in mice [32,33].

Although castor oil is most commonly recognized for its laxative and labor-inducing effects [16], its anxiolytic-like effect, shown here, is consistent with the therapeutic-like effects of other phytotherapies. For example, flax seeds [34] and olive oil [35] have antidepressant-like and anxiolytic-like effects. Like castor oil, olive oil is composed mainly of mixed triglyceride esters of oleic acid, palmitic acid, and other fatty acids, along with traces of squalene (up to 0.7%) and sterols (~0.2%; phytosterol and tocosterols). The anxiolytic-like effect of olive oil correlated with decreases in serotonin, the serotonin metabolite 5-hydroxyindoleacetic acid, and dopamine, but an increase in the dopamine metabolite homovanillic acid in the brain [35].

Like ricinoleic acid, the fatty acids palmitic acid [36], linoleic acid [37,38], and myristic acid [39], and fatty acids that are derived from other sources, such as fool’s parsley (*Aethusa cynapium*) [40], basswood (*Tilia americana* L. var. Mexicana) [41], and starflower (*Borago officinalis*) [42], have demonstrated anxiolytic-like effects. Moreover, palmitic and linoleic acids and numerous fatty acid analogs exert antidepressant-like effects [43,44,45]. Such antidepressant-like effects have also been shown for fatty acids that are derived from animal products, including omega-3 fatty acids and fish oils [46,47]. Previous research showed that anxiolytic-like effects of fatty acids were inhibited by picrotoxin but not by bicuculline or flumazenil, suggesting a response that is mediated by γ-aminobutyric acid-A receptors in the brain [48,49].

One hypothesis posits that neuroimmunological actions and neuroinflammation play critical roles in stress-related diseases [50]. The anti-inflammatory properties of castor oil and ricinoleic acid is consistent with this hypothesis [51]. Chronic topical treatment with ricinoleic acid for 8 days was shown to ameliorate carrageenan-induced edema of the mouse paw and histamine-induced edema of the guinea pig eyelid [52], although this study also found that the acute topical administration of ricinoleic acid (0.9 mg/mouse) before the insult caused an inflammatory effect [52]. Additionally, ricinoleic acid in a topical gel formulation markedly inhibited the synthesis of prostaglandin E_2_ [15]. Others found that oral Kolliphor administration caused antinociception in mice [53]. Furthermore, a comparative clinical trial demonstrated that castor oil effectively treated osteoarthritis, an inflammation-related disorder, similarly to the nonsteroidal anti-inflammatory drug diclofenac [13]. Future studies of mechanisms of action of ricinoleic acid, the active ingredient identified in our study, may provide new therapeutic avenues in phytotherapeutic science.

## 5. Conclusions

Our preliminary results suggest that castor oil and ricinoleic acid reduced anxiety-like behavior in male mice. The beneficial properties of castor oil, including wound healing, were known over 4000 years ago by ancient Egyptians and were published as early as the 18th century [2]. One potential limitation of castor oil use is its laxative, labor inducing, and potentially abortient effects when administered orally at high doses. In previous studies, transdermal castor oil administration had low transdermal absorption and no laxative effects [54] but was shown to exert anti-inflammatory and antinociceptive effects. Thus, follow-up studies could also investigate the anxiolytic-like and antidepressant-like effects of topical castor oil application. Our experiments also highlighted previously unknown effects of 5% Kolliphor, a vehicle that is commonly used in preclinical research, which slightly blunted the anxiolytic-like effect of chlordiazepoxide and slightly increased baseline open zone occupancy and entries. These data also highlight the importance of thoroughly investigating treatment vehicles that are used to study stress and related behaviors.

## Data Availability

The data used in this study are the property of the National Institute on Drug Abuse and can be provided upon request from the corresponding author.
